# Indo-Swiss partnership experience

**DOI:** 10.1186/1753-6561-7-S5-O2

**Published:** 2013-08-30

**Authors:** Mitchell G Weiss

**Affiliations:** 1 Swiss TPH, Switzerland

## 

The Indo-Swiss partnership has been based on the principles enunciated by the Swiss Commission for Research Partnership with Developing Countries. It provides support for scientific research projects, exchange of scientists and building partnerships for science.

The 11 Principles of Research Partnership have been elaborated under the following headings:

1. Decide on the objectives together

2. Build up mutual trust

3. Share information; develop networks

4. Share responsibility

5. Create transparency

6. Monitor and evaluate the collaboration

7. Disseminate the results

8. Apply the results

9. Share profits equitably

10. Increase research capacity

11. Build on the achievements

The Swiss Tropical and Public Health (Swiss TPH) aims to improve the health of populations at the national and international levels with an extensive network of activities in Africa, India and throughout the world. It collaborates and provides scientific support to the INDEPTH network (The International Network for the Demographic Evaluation of Populations and Their Health in Developing Countries), which comprises six sites in Asia, 12 sites in Africa and one site in Oceania. The INDEPTH network is involved in strengthening of Health and Demographic Surveillance Systems (HDSS). The Indo-Swiss symposium on cohorts and bio-banks with special reference to non-communicable diseases aims to consider and develop opportunities for Indo-Swiss partnership in research and to promote country and region-specific public health benefits.

